# Editorial: Factors determining long term anti-tumor responses to immune checkpoint blockade therapy

**DOI:** 10.3389/fimmu.2022.1120207

**Published:** 2022-12-21

**Authors:** Roberta Zappasodi, Graham P. Cook, Alison Taylor

**Affiliations:** ^1^ Weill Cornell Medicine, Cornell University and Memorial Sloan Kettering Cancer Center, New York City, NY, United States; ^2^ Leeds Institute of Medical Research, University of Leeds School of Medicine, Leeds, United Kingdom

**Keywords:** immunotherapy, tumour immunology, immune checkpoint blockade, checkpoint inhibitors, cancer

Landmark studies performed in animal models more than twenty years ago revealed that the anti-tumour activity of T cells could be enhanced by blocking inhibitory signals arising from cell surface receptors now collectively termed immune checkpoints (1–3). This led to the development of therapeutic antibodies targeting these molecules, their clinical trials and to the approval of agents targeting CTLA-4, PD-1 and its ligand, PD-L1 (3–5). These agents have revolutionised treatment of several cancers and are under investigation for their value in the treatment of many others. Two pioneers of this field, immunologists James Allison and Tasuku Honjo, were awarded the 2018 Nobel Prize in Medicine or Physiology (4, 5).

Using antibodies to treat cancer is not new. Antibodies targeting CD20 expression on B cell malignancies (Rituximab and its derivatives) and HER2 in breast cancer (Trastuzumab) have been approved for clinical use since the late 1990s. However, the antibody-mediated targeting of immune checkpoints represents a paradigm shift in cancer treatment. Along with agents targeting vascular endothelial growth factor (VEGF), these treatments target components of the tumour microenvironment rather than direct targeting of the tumour cells. This strategy reflects our changing view of cancer and the way in which it is studied and treated (6, 7). Attention is no longer confined to just the malignant cells, but now extends to all components of the tumour microenvironment and beyond (for example, the effects of co-morbidities and the microbiome). Numerous cell types are under investigation for their contribution to tumour progression and, hence, for their potential as drug targets; immune checkpoint blockade is a success story resulting from such endeavours.

Unfortunately, this success is not universal. In the case of melanoma, roughly half of the patients treated will not benefit. Furthermore, side effects can be severe. These therapies target naturally occurring feedback inhibitory mechanisms that have evolved to limit the duration of the immune response and minimise damage to healthy tissue. This is exemplified by early studies of PD-1 and CTLA-4 in which knockout mice were shown to develop autoimmune phenotypes. Side effects, variable responses (and of course cost) mean there is a pressing need to better understand the nature of response (and non-response) and to identify those patients more likely to benefit from these treatments. This has been intensive area of research for several years and the collection of papers presented here reflect clinical needs and current mechanistic understanding. The papers include those addressing immune checkpoint blockade in a single malignancy, as well as more diverse studies. Indeed, the impact of immune checkpoint therapy on cancer treatment is demonstrated by the number of cancer types investigated in these papers, these include melanoma, bladder, lung, renal, pancreatic and colorectal cancers. Furthermore, case reports in pancreatic cancer and rhabdomyosarcoma demonstrate how these therapies can dramatically improve outcomes for individual patients.

Several of these studies focus on defining markers of response to immune checkpoint therapy. A diverse range of material and technological approaches are used. For example, studies utilise tumour genome and transcriptome data, inflammatory markers (serum proteins and neutrophil to lymphocyte ratio), markers of circulating tumour cells and the contribution of the gut microbiome and tumour metabolism. In addition, factors that determine outcome are discussed, these include drug-drug interactions, as well as underlying immunological mechanisms and how the tumour microenvironment regulates and responds to treatment. Of note, these papers highlight the interdisciplinarity of these studies; biomedical researchers, oncologists and other clinical professionals now regularly work alongside bioinformaticians, statisticians, clinical trials experts and computer scientists. Most importantly, this field (which is no longer exclusive to immunologists) makes a difference.

## Author contributions

All authors listed have made a substantial, direct, and intellectual contribution to the work and approved it for publication.
